# Targeting blood-derived IL-1 to treat brain injury

**Published:** 2013-07

The inflammatory mediator interleukin-1 (IL-1), which is known to be a driver of chronic diseases such as diabetes and hypertension, has recently been identified as a major contributor to ischaemic brain injury. IL-1 is made by the haematopoietic (blood) system and also by cells in the brain. The relative contributions of central versus peripheral sources of IL-1 to brain injury have not been previously investigated. Here, Stuart Allan and colleagues selectively eliminated haematopoietic-derived IL-1 in a chimeric mouse model of ischaemic brain injury. They demonstrate that both sources of IL-1 are important for disease development, but removal of the peripheral source alone is sufficient to improve neurological outcome in the mice. This suggests that therapies that target peripheral IL-1 could be beneficial in brain injury, potentially overriding the need to deliver drugs across the blood-brain barrier. **Page 1043**

